# Latissimus dorsi muscle flap transfer through endoscopic approach combined with the implant after tissue expansion for breast reconstruction of mastectomy patients

**DOI:** 10.1186/s12893-021-01464-0

**Published:** 2022-01-08

**Authors:** Jian-Xun Ma, Bi Li, You-Chen Xia, Wei-Tao You, Jie Zhang, Yi-Mou Sun, Xu Chang, Yue Lang

**Affiliations:** Department of Plastic Surgery, Peking University Third Hospital, #49, North Garden Road Haidian District, Beijing, 100191 People’s Republic of China

**Keywords:** Endoscopic technique, Latissimus dorsi muscle, Implant, Two-stage, Breast reconstruction

## Abstract

**Background:**

Implant-based breast reconstruction is easy to be performed but has flaws that an unnatural appearance might be presented when no sufficient coverage existing. While autologous tissue reconstruction also has disadvantages like donor site scar and skin patch effect. There is a demand for a new method to obtain natural and aesthetic appearance while surmounting drawbacks of conventional breast reconstruction surgery.

**Methods:**

A retrospective review of thirty-one patients undergoing tissue expander (TE)/implant two-stage breast reconstruction with latissimus dorsi muscle flap (LDMF) transfer through endoscopic approach in Peking University Third Hospital from April 2016 to August 2020 was performed. The LDMF harvest time, drain time, and complications were reviewed. The 3D volume was obtained to assess the volume symmetry of bilateral breasts. The BREAST-Q reconstruction module was used to evaluate the satisfaction.

**Results:**

The mean endoscopic LDMF harvest time was 90.4 min. In the mean follow-up of 11.2 months, there were no severe capsular contracture happened. The reconstructed side achieved good volume symmetry to the contralateral side (P = 0.256). Based on the evaluation of the BREAST-Q scores, the outcome of Satisfaction with Breasts was excellent or good in 87.1% of the cases.

**Conclusions:**

The novel type of two-stage breast reconstruction protocol, which includes tissue expansion followed by implant insertion with endoscopy-assisted LDMF transfer, could effectively reduce visible scars, avoid the patch effect, while require short time for LDMF harvest and present low incidence of complications. It is a promising method for breast reconstruction because it achieves good outcomes in the mastectomy patients.

## Background

The number of patients suffering from breast cancer is increasing in China [1]. The deficiency of breasts by mastectomy greatly influences women’s normal lives, thus the surgery of breast reconstruction is now much in demand.

There are various methods of breast reconstruction for choice. All the procedures are used to supply the excessive skin envelop and compensate for the breast tissue loss. As for implant-based reconstruction, there are often criticisms that no sufficient soft tissue coverage on the prosthesis, especially after tissue expansion, cause an unnatural appearance [2]. As for autologous tissue reconstruction, the latissimus dorsi myocutaneous flap has been a workhorse of reconstructive surgery, but it is always used for the reconstruction of small-to-medium breast [3, 4]. Moreover, the conventional harvest technique requires an obvious back incision that can be between 15 and 45 cm in length, in addition to an axillary incision for pedicle dissection or flap transfer. In addition, there is a trend that patients do not favour a skin patch on the surface of reconstructed breast. Nowadays, endoscopy-assisted techniques are being applied throughout plastic surgery, which makes it possible to harvest the latissimus dorsi muscle flap (LDMF) through a minimal invasive approach.

In consideration of overcoming those drawbacks of sole prosthesis and latissimus dorsi myocutaneous flap breast reconstruction, we innovatively combine endoscopic LDMF transfer with tissue expander (TE) /implant two-stage breast reconstruction. Through this new type of breast reconstruction protocol, the long prominent scar on the back could be avoided, and the coverage over the prosthesis could also be enhanced. The purpose of this study is to investigate the aesthetic outcomes in a single surgical group practice and to evaluate its safety and effectiveness.

## Methods

### Patients

The patients undergoing TE/implant two-stage breast reconstruction in our department from April 2016 to August 2020, in which endoscopy-assisted LDMF transfer was conducted on the second stage, were included in this study. The inclusion criteria for patient selection are defined as ① female ≥ 20 years old and ≤ 55 years old; ② patients underwent unilateral mastectomy because of breast cancer; ③ existence of LDM and thoracodorsal artery confirmed by ultrasound. The exclusion criteria are defined as ① poor function of ipsilateral LDM; ② the contralateral breast has severe ptosis; ③ other contraindications for LDMF or implant surgery.

This study was approved by Peking University Third Hospital Medical Science Research Ethics Committee (No. M2018278). Informed consent was obtained from all patients. All methods performed in this study were in accordance with the Declaration of Helsinki.

### Surgical technique

On the first stage of breast reconstruction, the pectoralis major muscle and the serratus anterior muscle near the anterior axillary line are elevated from the chest wall via the mastectomy incision. Then a 400 ml round TE (Guangzhou Wanhe Plastic Materials Co., Ltd., Guangzhou, China) is inserted into the sub-pectoral and sub-serratus pocket followed by setting a closed suction drainage (Suzhou Kebang Polymer Medical Apparatus Co., Ltd., Suzhou, China) in the pocket, and the edges between pectoralis major muscle and serratus muscle are sutured together to close the pocket. The volume of saline injected into the TE is approximately 25% of the total volume of the expander, unless the mastectomy flaps or the muscle pocket do not look healthy or strong enough to tolerate this. The skin incision is closed at last.

One week after operation, the expansion could begin if no signs of ischemia and necrosis of flaps are observed. The volume of water injection is usually 10% of the expander capacity with interval of one week between inflations. The expansion finishes when the volume of the affected side compared to the contralateral side reaches about 160% [5].

**Fig. 1:**
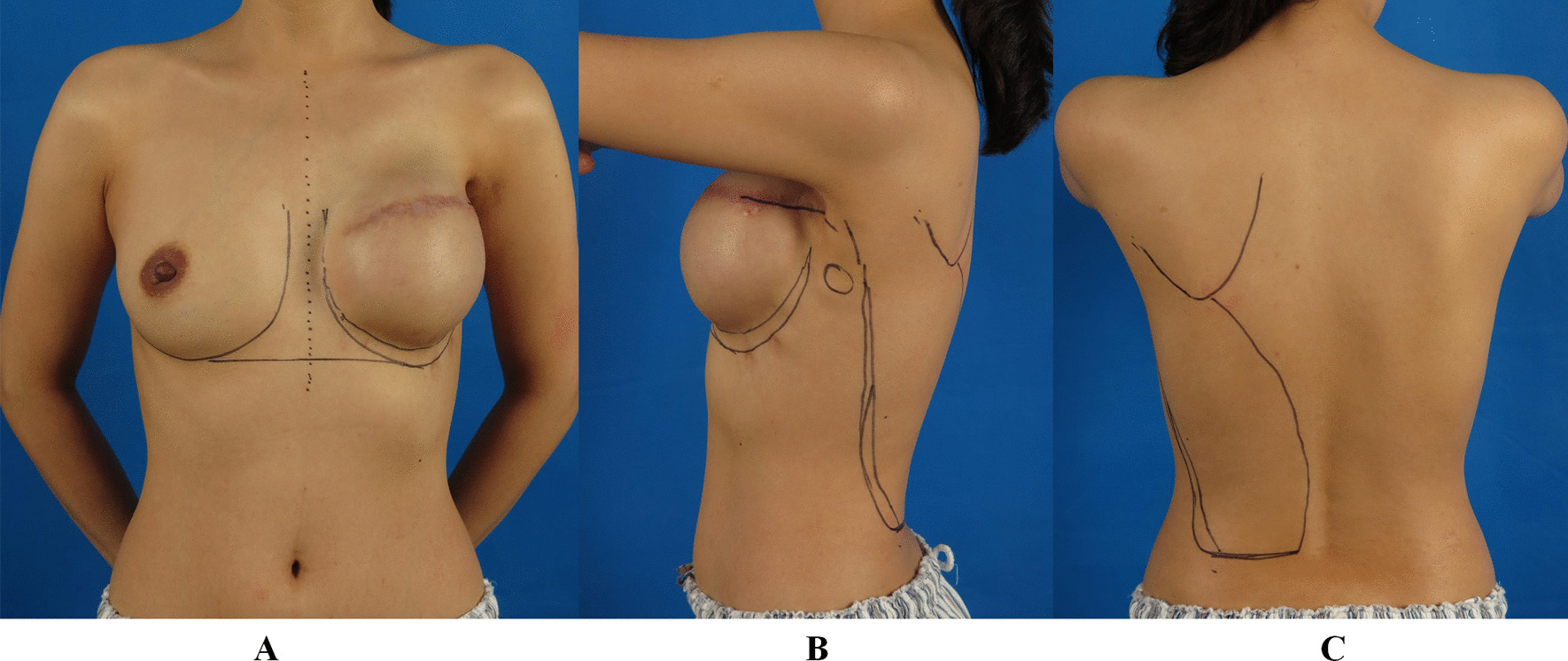
Marking before the endoscopic LDMF transfer combined with implant insertion on the second stage of breast reconstruction. **A** The midline, borders of bilateral breasts, and inframammary fold are marked on the chest wall. **B** The mastectomy incision extending to the posterior axillary fold is marked as the endoscopic approach. **C** The borders of the latissimus dorsi muscle are marked on the back: the anterior border can be palpated during active muscle contraction, the superior border is marked from the tendinous insertion along the tip of the scapula to the medial border along the paravertebral origin, and the inferior margin is marked at the iliac crest. *LDMF* latissimus dorsi muscle flap

On the second stage, when the tissue expansion is finished, markings are prepared preoperatively while the patient is in an upright position. The markings include the midline, inframammary fold, lateral edge of breast tissue on the chest wall, and the lateral margin of the LDM along the posterior axillary line, inferior margin at the iliac crest, medial margin along the paravertebral origin, superior margin at the tip of the scapula, and also the incisions (Fig. 1).

**Fig. 2:**
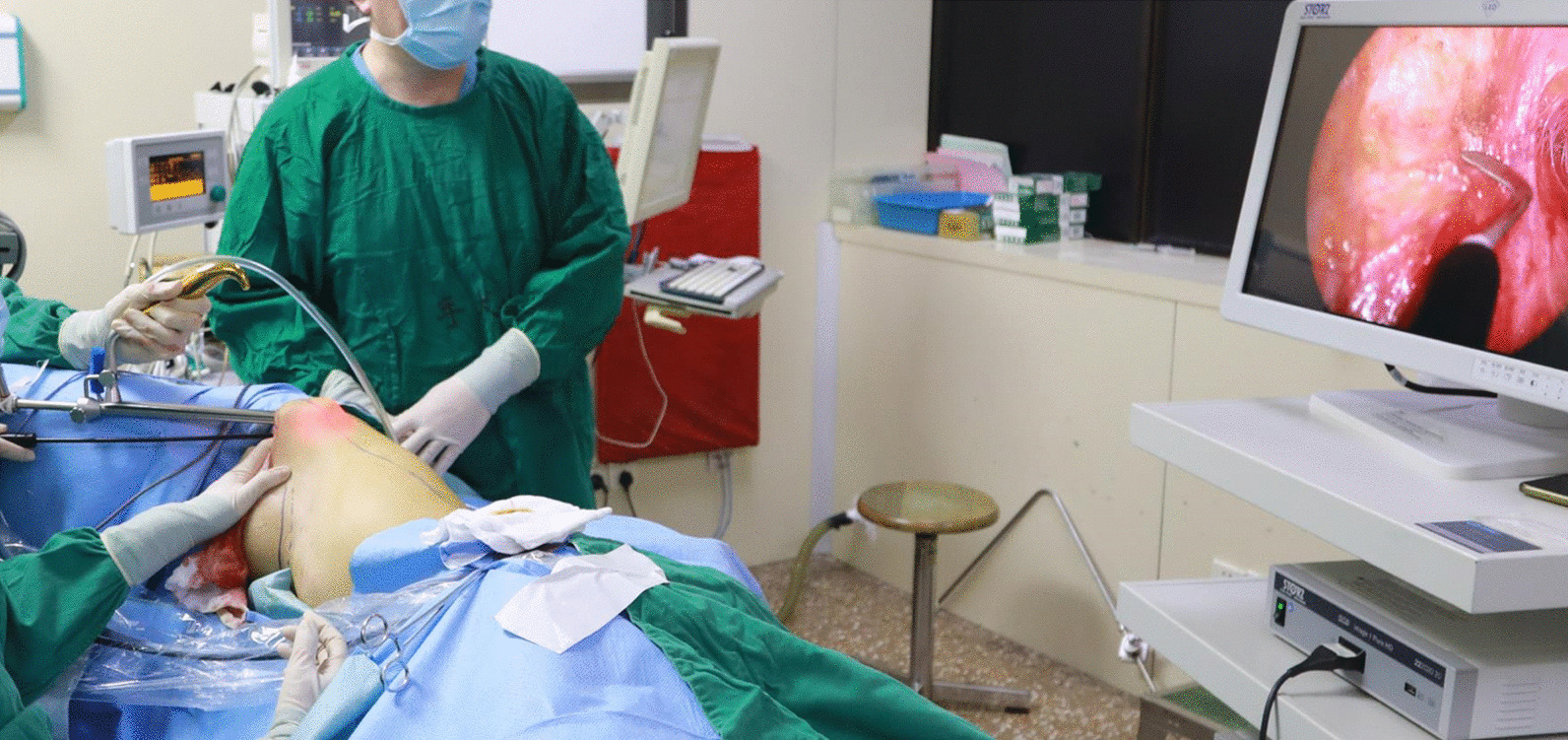
Endoscopic approach to achieve the harvest of LDMF. The previous mastectomy incision extending to the posterior axillary fold is used as the approach to perform the endoscopy-assisted LDMF harvest. The subcutaneous plane is dissected first by a long monopolar electrosurgical hook. The endoscopic procedures are performed under the view of a video monitor. LDMF: latissimus dorsi muscle flap

During the operation, the patient is placed in a lateral decubitus position with the relevant upper extremity abducted and rested on a supporter. The previous mastectomy incision extending to the posterior axillary fold is opened through the skin. The TE is identified and removed. In the meanwhile, the superior anterior border of LDM is exposed as surgeons continue the macroscopic subcutaneous and submuscular dissection to create an initial optic cavity, which serves as a landmark for subsequent endoscopic muscle dissection. The tumescent solution including 250 ml Lactated Ringer, 1 mg adrenaline, and 200 mg lidocaine, is injected into both subcutaneous and submuscular planes. Then a 10-mm-diameter rigid endoscope with a 30-degree-angle lens (KARL STORZ Gmbh & Co. KG, Germany) is set up together with a U-shaped retractor (Shanghai Zhonghetiangong Medical Instrument Co., Ltd., Shanghai, China). The retractor and endoscope inserted through the incision are held by an assistant to maintain an optical cavity that facilitates the dissection (Fig. 2). The retractor which has holes in the tip is connected with vacuum suction in order to suck out the cautery smoke and heat. The subcutaneous plane is dissected first by a long monopolar electrosurgical hook (Shanghai Zhonghetiangong Medical Instrument Co., Ltd., Shanghai, China), then the dissection is carried out along the undersurface of the muscle. The endoscopic procedures are performed under the view of a video monitor. The thoracodorsal vessels are identified underneath the LDM with the aid of an L-shaped fiber-optic retractor (Changzhou Jinyang Medical Instrument Co., Ltd., Changzhou, China) under direct vision, and the pedicle is marked with a vessel loop which makes it easy to ensure its safety. When the dissection is completed on both the superficial and deep planes, an additional 4-cm-long transverse incision is applied on the posterior waist under the level of the posterior superior iliac ridge. The muscle is disinserted from the paravertebral origin through both the axillary and posterior lumbar incision under the endoscopic view, and the inferoposterior part dissection is also facilitated by the approach of the lumbar incision. The muscle then can be easily pulled out through the initial incision without tension and rotate to the recipient pocket temporarily. The donor site is checked and hemostasis is conducted with the aid of endoscopy, and a closed suction drainage is applied through the lumbar incision followed by the closure of this incision.

**Fig. 3:**
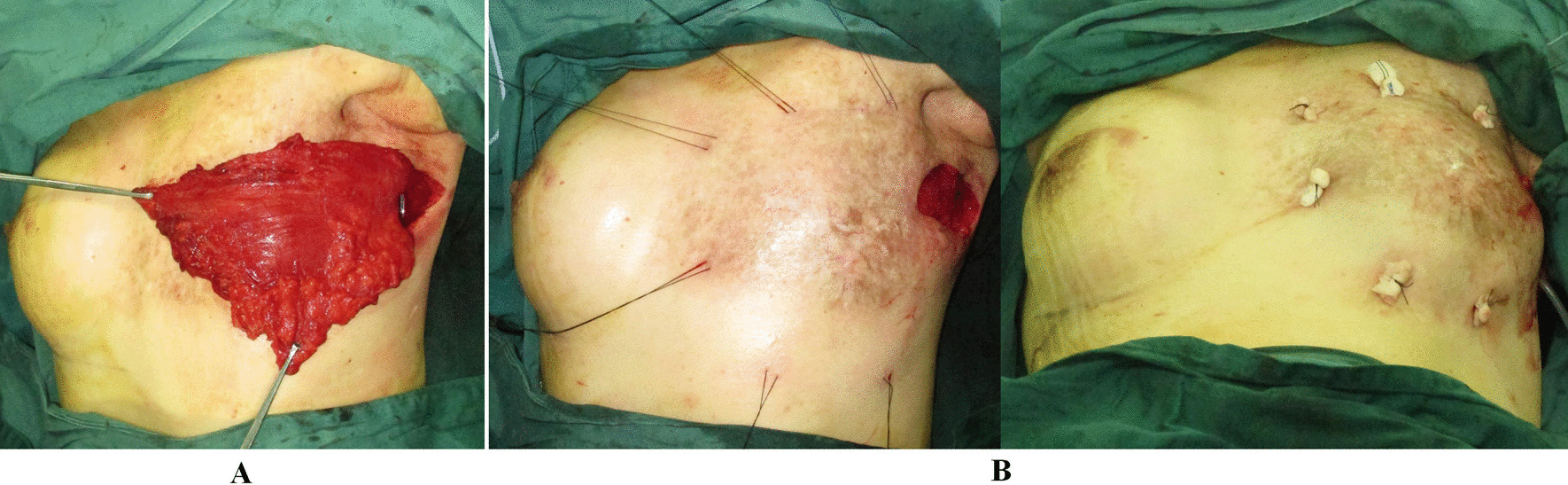
The transfer of LDMF as an additional cover for the final implant. **A** LDMF is harvested using endoscopic technique through previous mastectomy incision approach and rotates to the affected chest wall. **B** The muscle flap is inserted into the expanded pocket, and the edges are fixed to the pocket through external bolster sutures, which serve as a complete cover for the implant. The bolster made of thirty-two layers of Vaseline gauze measuring 1.0 cm in length and 0.5 cm in width is secured by mattress suture of 0 silk suture. *LDMF* latissimus dorsi muscle flap

The patient is shifted to the supine position. A capsulectomy is performed if necessary, and the pocket is irrigated with 60 ml saline plus gentamicin 80,000 IU. The LDMF is inserted into the pocket, and the edges are fixed to the medial, inferior, and lateral aspects of the pocket through 4-6 external bolster sutures, which serve as a complete cover for the implant (Fig. 3). The bolster made of thirty-two layers of Vaseline gauze measuring 1.0 cm in length and 0.5 cm in width is secured by mattress suture of 0 silk suture (ETHICON®, Johnson & Johnson Medical Ltd., Shanghai, China). Then the patient is kept in the sitting position, and a breast implant sizer (MENTOR®, Mentor Medical System B.V., Leiden, Netherlands) is routinely used to facilitate the determination of final implant size and location. A closed suction drainage is introduced in the implant pocket followed by the permanent implant (MENTOR®, Mentor Medical System B.V., Leiden, Netherlands) placement under the LDMF. The skin incision is closed at last.

The drain tubes are removed when the output of each is less than 20 ml per day postoperatively. Cotton pads with elastic bandages are applied for two weeks and an elastic vest is to be worn for two more weeks. Appropriate immobilization of the relevant shoulder joint in functional position for two weeks is recommended. Afterwards, the shoulder movement range should be restored gradually.

### Acquisition of the three-dimensional surface image

Three-dimensional surface image (3D-SI) is acquired by VECTRA-XT stereophotogrammetry device (Canfield Scientific Inc, Fairfield, NJ, USA). Breast Sculptor® software (version 5.5.3, Canfield Scientific Inc, Fairfield, NJ, USA) is used to provide breast measurement data. When the breast boundaries are determined, the breast volume can be calculated automatically after the virtual chest wall is simulated from the landmarks by the software [5]. The 3D images of every patient were acquired at more than 6 months post-reconstruction follow-up visits.

### Evaluation of patient satisfaction

An evaluation of patient reported outcomes was conducted by utilizing BREAST-Q Reconstruction Module Postoperative Scales Mandarin Version. The scales included Satisfaction with Breasts. The satisfaction was evaluated as follows [6]: excellent (score 81–100), the bilateral breasts were highly symmetrical, and the patient was highly satisfied; good (score 61–80), the reconstructed breast was a little asymmetrical with the contralateral side, and the patients was satisfied; fair (score 31–60), the reconstructed breast was asymmetrical with the contralateral breast, and the patient was not satisfied; and bad (score 0–30), the reconstructed breast presented distortion.

### Statistical analysis

Breast volume asymmetry is assessed using the following formula: [right breast volume−left breast volume]/the larger breast volume × 100%, which represents the volume difference between the two breasts [7]. The data are analyzed using paired-sample T test. The measurement data are expressed as mean ± standard deviation. P values are two-tailed, and P values < 0.05 are considered significant. All the data are analyzed using IBM SPSS Statistics 25.0 (International Business Machines Corp., USA).

## Results

### Patients

Thirty-one patients were included in this research. The average age was 37.6 years (26.0 to 50.0 years), and the average body mass index (BMI) was 21.4 kg/m^2^ (19.2 to 29.7 kg/m^2^). The TNM anatomic stages of breast cancer were I and II. The final pathological outcomes of the mastectomy specimens were invasive ductal carcinoma and ductal carcinoma in situ. Twenty-eight patients received chemotherapy. Two patients underwent radiation treatment at the end of tissue expansion and received LDMF transfer six months after the radiation, and one patient received radiation post-mastectomy and was applied delayed TE insertion. The demographic data are shown in Table 1.

**Table 1:** Demographic data of thirty-one patients undergoing endoscopy-assisted LDMF transfer combined with implant insertion after tissue expansion for breast reconstruction

Category (n = 31)	Mean ± SD / n(%)
Age	37.6 ± 6.4
Body mass index (kg/m^2^)	21.4 ± 2.3
TNM anatomic stage (breast cancer)	
Stage I	13 (41.9%)
Stage II	18 (58.1%)
Final pathology	
Invasive ductal carcinoma	24 (77.4%)
Ductal carcinoma in situ	7 (22.6%)
Affected side	
Right	13 (41.9%)
Left	18 (58.1%)
Chemotherapy	
Yes	28 (90.3%)
No	3 (9.7%)
Radiation	
Yes	3 (9.7%)
No	28 (90.3%)

*LDMF* latissimus dorsi muscle flap

### Reconstruction process

Ten patients were treated with immediate TE placement, and twenty-one patients were provided with delayed TE insertion. The average time of LDMF harvest using the endoscopic technique was 90.4 min (70.0 to 120.0 min). The average length of LDMF was 21.4 cm (18.0 to 27.0 cm), and the width was 13.8 cm (12.0 to 18.0 cm). The mean volume of the final implant was 228.7 ml (175.0 to 315.0 ml). The contralateral symmetry surgery included breast augmentation with an implant for six patients (three on the first stage and three on the second stage), breast augmentation with autologous fat grafting for four patients (three on the first stage and one on the second stage), and mastopexy for three patients (one on the first stage and two on the second stage). The clinical data about the reconstruction process are shown in Table 2.

**Table 2:** Clinical data during the reconstruction process

Category (n =31)	Mean ± SD / n(%)
Reconstruction timing	
Immediate	10 (32.3%)
Delayed	21 (67.7%)
LDMF harvest time (min)	90.4 ± 13.7
LDMF length (cm)	21.4 ± 2.4
LDMF width (cm)	13.8 ± 1.4
Final implant volume (ml)	228.7 ± 34.2
Contralateral symmetry surgery	
Augmentation with an implant	6 (19.4%)
Augmentation with fat grafting	4 (12.9%)
Mastopexy	3 (9.7%)
None	18 (58.0%)

*LDMF* latissimus dorsi muscle flap

### Post-reconstruction

**Fig. 4:**
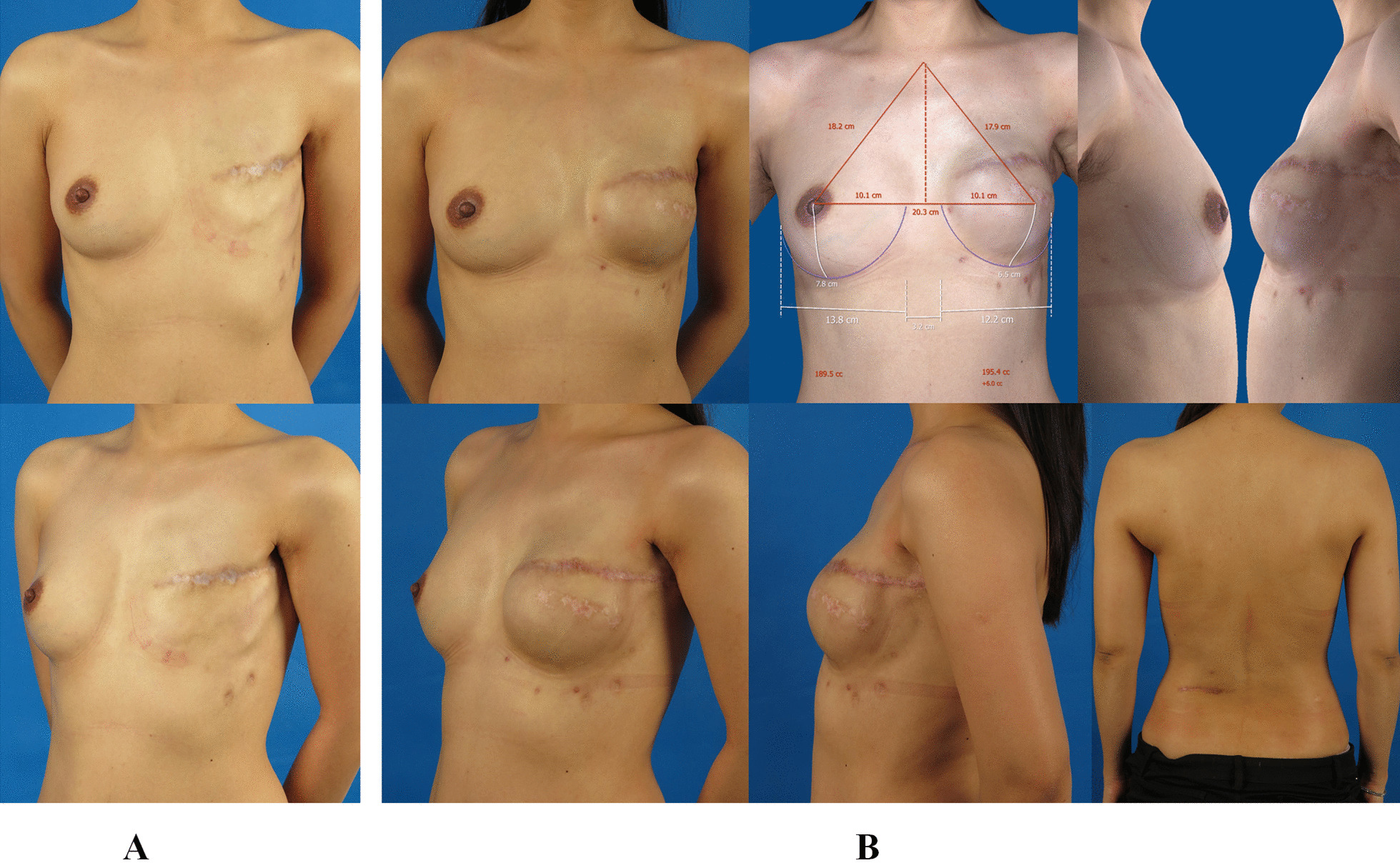
The outcome of tissue expansion/implant with endoscopy-assisted LDMF transfer two-stage breast reconstruction at thirteen-month follow-up. **A** A 33 years old woman received left modified radical mastectomy because of breast cancer. The frontal and oblique pictures of the affected side were presented. **B** The patient underwent implant insertion with endoscopy-assisted LDMF transfer on the second stage of left breast reconstruction following tissue expansion on the first stage. The frontal, lateral and oblique pictures of the reconstructed breast were obtained at thirteen-month follow-up. The 3D image indicated the two breasts achieved good volume symmetry. The back view showed the scar of the lumbar incision was not obvious. *LDMF* latissimus dorsi muscle flap

**Fig. 5:**
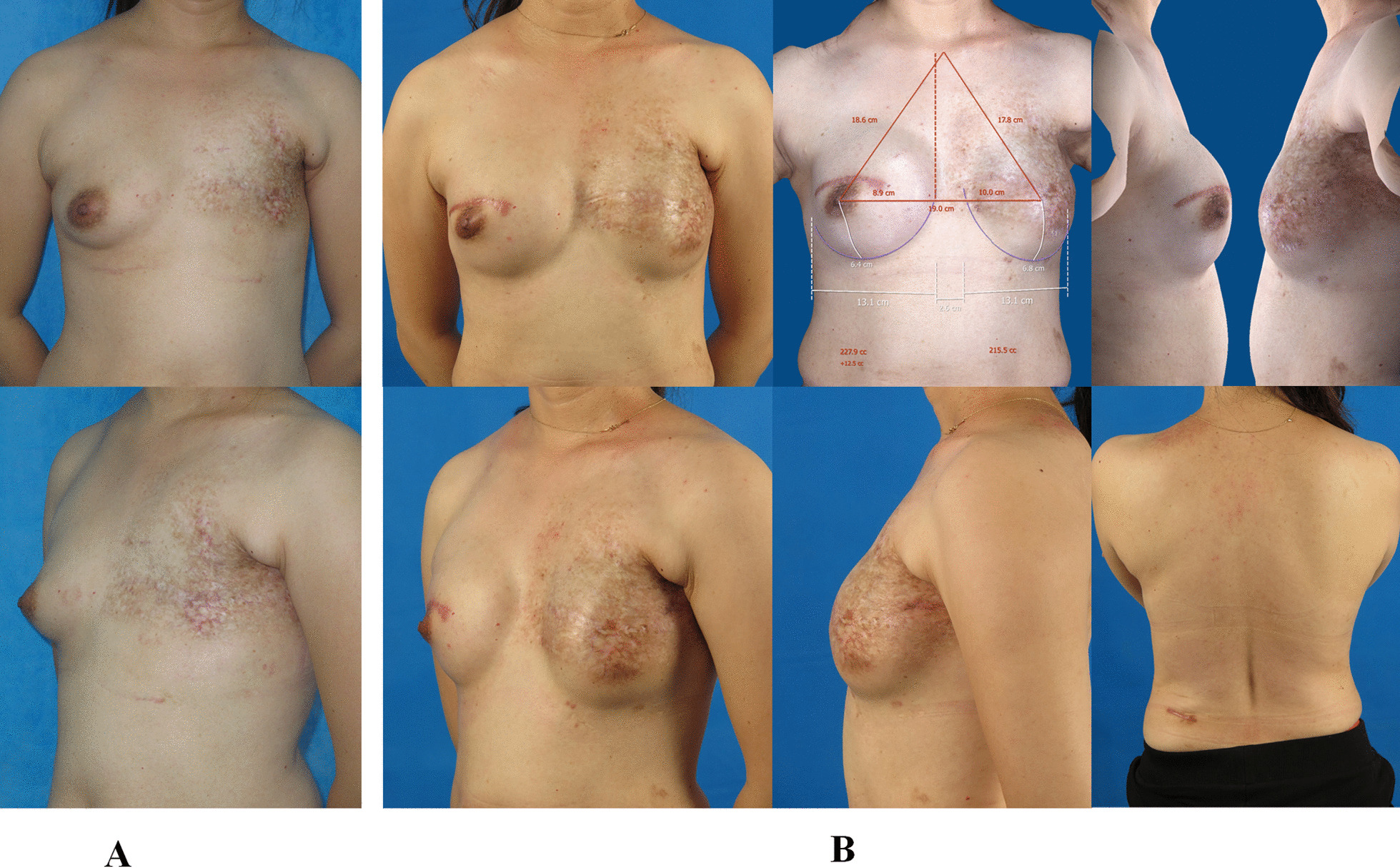
The outcome of the patient who was applied tissue expansion/implant with endoscopy-assisted LDMF transfer two-stage left breast reconstruction combined with implant augmentation of right side at twelve-month follow-up. **A** A 33 years old woman underwent modified radical mastectomy for left breast cancer, then she received radiation therapy. The frontal and oblique pictures of the affected side were presented. **B** The patient received right nipple-sparing mastectomy because of fibroadenomatoid adenosis, followed by right breast augmentation with an implant, in the meanwhile, she was applied a tissue expander insertion on the first stage of left breast reconstruction. When the tissue expansion was finished, the patient underwent implant insertion with endoscopy-assisted LDMF transfer on the second stage of left breast reconstruction. The frontal, lateral and oblique pictures of the reconstructed breast were obtained at twelve-month follow-up. The 3D image indicated the two breasts achieved good volume symmetry. The back view showed the additional incision could be concealed when wearing pants. *LDMF* latissimus dorsi muscle flap

**Fig. 6:**
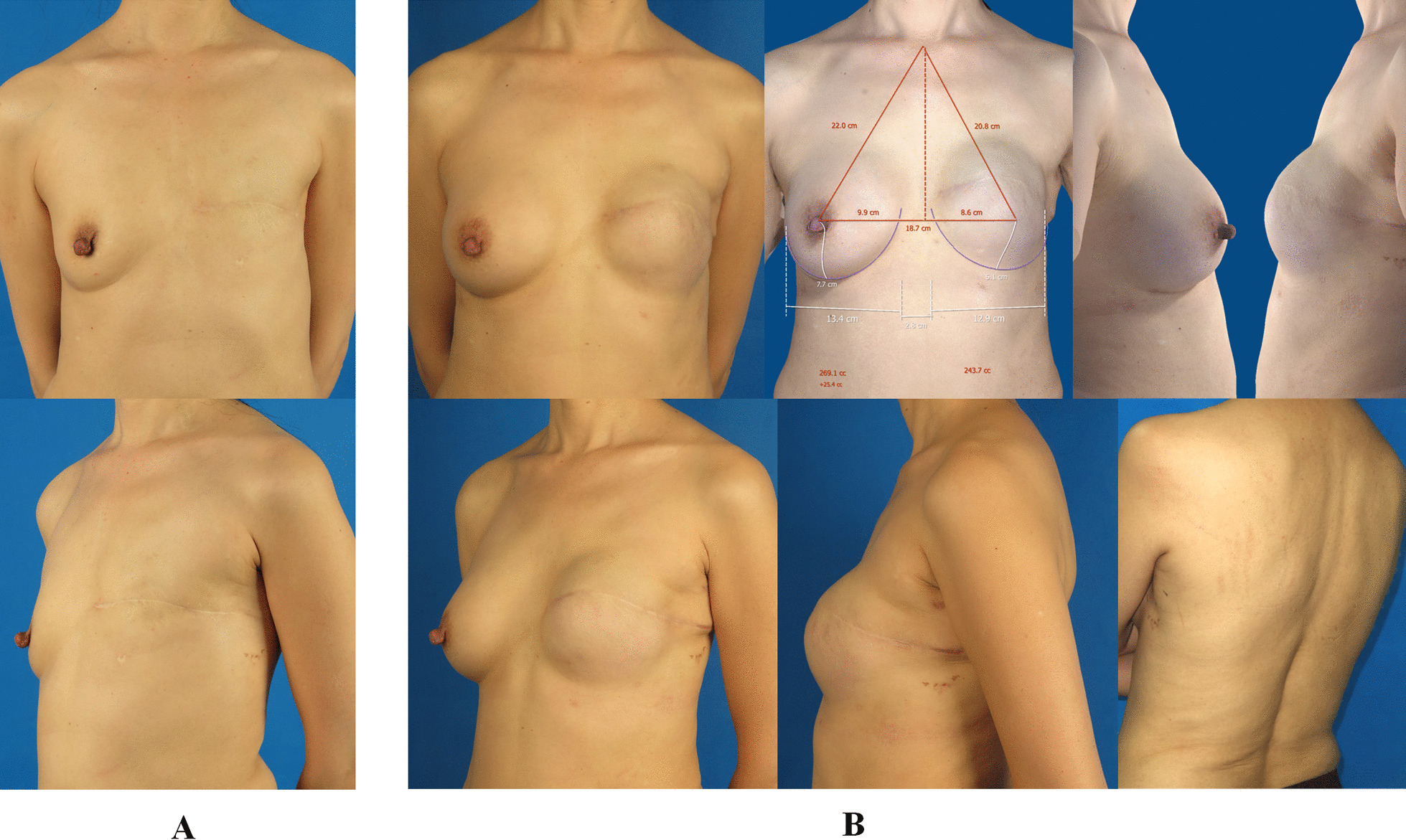
The outcome of the patient who was applied tissue expansion/implant with endoscopy-assisted LDMF transfer two-stage left breast reconstruction combined with implant augmentation of right side at twenty-month follow-up. **A** A 50 years old woman underwent modified radical mastectomy for left breast cancer. The frontal and oblique pictures of the affected side were presented. **B** The patient underwent implant insertion with endoscopy-assisted LDMF transfer on the second stage of left breast reconstruction following tissue expansion on the first stage. In the meanwhile, she underwent right breast augmentation with an implant. The frontal, lateral and oblique pictures of the reconstructed breast were obtained at twenty-month follow-up. The 3D image indicated the two breasts achieved good volume symmetry. The back view showed the scar of the lumbar incision was almost invisible. *LDMF* latissimus dorsi muscle flap

The average drain time was 10.1 days (6 to 19 days). Four patients were found seroma formation, who were treated with aspiration. No other complications like hematoma, or wound healing abnormality were observed. All the patients got a follow-up from 6 months to 28 months (mean 11.2 months) post-reconstruction, and 3D images were acquired. Ten patients had a clinical capsular contraction with the Baker classification IB, sixteen patients with class II, five with class III, and no severe capsular contracture happened [8]. The average 3D-volume of the reconstructed breasts was 246.9 ml (182.8 to 327.8 ml), the average 3D-volume of the contralateral breasts was 250.2 ml (177.6 to 340.2 ml), and the average breasts volume asymmetry post–reconstruction was 4.8% (2.3–12.3%). There was no significant difference between reconstructed and contralateral breasts (P = 0.256), which indicated that the bilateral breasts achieved good volume symmetry after reconstruction (Table 3). Twenty-nine patients finished the questionnaire of BREAST-Q at average 18 months post-reconstruction, and two patients did not complete it due to personal reasons. The average post-operative score of Satisfaction with Breasts was 69.1. Based on the evaluation of the BREAST-Q score, the outcome of Satisfaction with Breasts was excellent in five patients, good in twenty-two patients, and fair in two patients. Thus, the outcome was excellent or good in 87.1% of the cases (Table 4). Pre-/post-operative photographs and 3D images of three patients are shown in Figs. 4, 5 and 6.

**Table 3:** Clinical data post-reconstruction

Category (n = 31)	Mean ± SD / n(%)	P
Drain time (day)	10.1 ± 3.5	
Follow–up (month)	11.2 ± 4.9	
Complications		
Seroma	4 (12.9%)	
None	27 (87.1%)	
Volume of reconstructed breast (ml)	246.9 ± 42.1	0.256
Volume of contralateral breast (ml)	250.2 ± 45.6	
Breasts asymmetry (%)	4.8 ± 2.4	

*P* paired-sample T test

**Table 4:** BREAST-Q reconstruction module Satisfaction with Breasts scale scores

Score	n(%)	Mean ± SD
81–100 (Excellent)	5 (16.1%)	
61–80 (Good)	22 (70.9%)	
31–60 (Fair)	2 (6.5%)	
0–30 (Bad)	0	
None^a^	2 (6.5%)	
Average		69.1 ± 10.3

The questionnaire was completed average 1.5 years after reconstruction

^a^Patients who did not complete the questionnaire due to personal reasons

## Discussion

Breast reconstruction is considered as a part of breast cancer treatment to restore breasts to their near-normal shape, size, and symmetry, and to enhance the quality of patients’ lives. Nowadays, the demand for breast reconstruction is increasing constantly, which leads plastic surgeons to look for new methods to obtain a more natural and aesthetically pleasing appearance of the reconstructed breasts.

Because a large amount of skin and a considerable volume of inner tissue have been lost due to mastectomy, the restoration of skin envelope and the compensation for the breast tissue loss are two key issues that need to be considered during reconstructive surgery. In fact, tissue expansion two-stage reconstruction, which could expand the pectoralis major muscle and its overlapped skin at the same time, so as to restore the soft tissue cover, has become the most popular reconstruction method in China. Then volume replacement techniques, like implants insertion or distant autologous tissue flaps transfer, could help to compensate for the tissue loss. Among these, the implant is one of the most frequently used for most Chinese patients. Yet the implant always makes the breast feel stiff and the transition area between breast and chest wall looks unnatural, especially when the implant size is large to fit the large contralateral breast. As for the autologous tissue flap, mastectomy with LD flap transfer could only be beneficial for women with small-to-medium-sized breasts with upper outer quadrant breast cancer who desire breast conservation surgery [3, 4, 9], because the provided volume is insufficient to achieve the total breast reconstruction.

In this study, we designed a new two-stage breast reconstruction protocol, which included tissue expansion on the first stage and implant insertion combined with LDMF transfer on the second stage. Using this method, the total extension of the pectoralis major muscle together with the transferred LDMF provide a robust muscular cover over the permanent implant, which makes the breast feel soft and smooth the transition region between the breast mound and the chest wall. Moreover, the additional volume of the LDM adding to the reconstructed breast allows choosing a small-sized implant, which also enhances the touch feeling of the breast.

Feng et al. [10] reported a similar two-stage breast reconstruction protocol, but the difference was that the LDMF transfer was applied together with the tissue expander insertion on the first stage. Regarding tumor staging, it is important to identify patients who will require adjuvant radiation therapy. Under this circumstance, the LDMF should be preserved as a salvage option for use in a delayed reconstruction setting. Because the non-irradiated autologous tissue flap could provide non-irradiated cellular elements which can lead to the repair of the dermal fibrosis resulted from post-mastectomy radiation therapy [11]. Moreover, it was also showed that the harvest of the LDMF is associated with a low complication rate and reliable results for delayed reconstruction of the irradiated breast [12, 13]. So, under consideration of the uncertainty about the application of radiation therapy in the immediate reconstruction cases, we leave the LDMF transfer on the second stage for breast reconstruction in this difficult to predict clinical scenario.

Previously, the breast envelope often needs to be enlarged with an LD skin paddle to maximize the size of the implant in a single-stage reconstruction. However, this leads to a skin patch presented on the reconstructed breast which does not match the color, texture, or thickness of the native breast skin [12]. In our new breast reconstruction protocol, tissue expansion and the use of endoscopic assistance in muscle harvest are operative modalities designed to avoid the cutaneous patch effect of the transposed musculocutaneous paddle, which results in no skin mismatch from the donor to the recipient site.

The conventional LD flap harvest requires a long incision that often results in an apparent scar over the back which becomes one of the main concerns of the patients [9]. The endoscopy-assisted muscle harvest technique is becoming popular in breast reconstruction because it obviates the need for an obvious posterior donor site scar by using a small lateral extension of the mastectomy incision.

Many techniques for creating the optical cavity for endoscopic operation have been described already. Some authors preferred manual traction with endoscopic retractors [14–16] or operated with the aid of traction stitches in the skin [17], while others favored gas inflation using trocars to fit the laparoscopic instruments [4, 18] or robotic arms [3, 12, 19, 20]. All these procedures achieved harvest of the LDMF with fewer visible scars than the conventional technique. Nevertheless, all of them created a vertical scar or three to four incisions vertically lined along the side of the chest for insertion of the trocars. Although a vertical incision is much easier to harvest the LDMF [16], a transverse incision is preferred for its better aesthetic outcome. Unlike setting the incision or multiple ports cut along the vertical posterior axillary line in other reports, we apply a transverse incision extending along the mastectomy scar, which enables the incision to be concealed under the axillary fold.

Dividing the LDM from its paravertebral origin and iliac attachments is the challenging part of the procedure, because of the narrow operative view and the difficulty in the resection of the distant LDM over the thorax anatomic curvature. Moreover, it is hard to control the bleeding during the medial dissection encountering the lumbar perforators, precisely where the access is most restricted. Some studies reported techniques for LDMF harvest via the mastectomy incision and axillary incision under the endoscopic guidance [14, 21]. Although these techniques have the advantage of no scar on the back, various specific retractors and other self-designed instruments are essential for the procedure. Moreover, these techniques require rich endoscopic surgery experience and a long learning curve to achieve efficiency and safety, which are quite unfriendly to beginners.

We add one more approach for endoscopy-assisted LDMF harvest to balance the difficulty of the procedure and the complication issues against the negative effects of longer scars. In this study, an additional short incision was applied on the posterior waist under the level of the posterior superior iliac ridge to facilitate the LDMF harvest, which made it easy to divide the inferoposterior origin of the muscle and perform the hemostasis. Also, this incision can be concealed perfectly when wearing pants.

The endoscopic technique does have a learning curve because of the lack of tactile sensation when using the long endoscopic instruments and the lack of depth nature from the two-dimensional video screen. Therefore, endoscopy-assisted LDMF harvest does require prolonged surgical time. Although various techniques had been employed to facilitate this procedure, the average LDMF harvest time was still around 120 ~ 240 min [4, 18, 22]. Even the latest da Vinci robotic technique required over 120 min [19, 23]. The mean LDMF harvest time in this study was 90.4 min, which was much shorter than previous studies. The short duration was attributed to the aid of the supplementary posterior lumbar approach. To be sure, the endoscopy-assisted harvest time can also decrease once the learning curve is overcome by the constant use of this technique.

Previous studies have demonstrated that this minimal invasive LDMF harvest technique can be performed with a lower complication rate compared with traditional open techniques [12]. And the most common complication of breast reconstruction with LD flap is donor site seroma [24]. There was 12.9% seroma occurrence rate postoperatively in this study, which is better than the 28.6% seroma formation rate in a research including 14 cases undergoing endoscopic LDMF harvest for breast reconstruction [4]. The good result might be attributed to the prolonged periods of drainage, elastic compression of the operation areas, and immobilization of the shoulder joints. Compared with 8.0 days in a recent study applying endoscopy-assisted LDMF harvest for breast reconstruction [21], the mean drain time in our research was 10.1 days. As for the reason, the strict criteria for drain removal was considered (less than 20 ml VS 50 ml per day). A longer time of drain tubes maintenance is prone to cause the relevant complications like secondary infection or wound dehiscence, so the meticulous medical care is essential.

There were several limitations in this study. First, the sample size of this research was small. Therefore, further studies involving a larger number of patients are needed to evaluate the effectiveness and safety of this type of breast reconstruction protocol. Second, although the mean follow-up time reached 11.2 months, it is not long enough to reveal unpleasant complications like capsular contracture which might occur many years after the surgery. Additionally, the final outcomes were evaluated only by the volume symmetry, while the contour symmetry including base width and projection are also important indicators to be assessed, which is believed to be involved in the subsequent researches. Despite the limitations, early success in this type of breast reconstruction protocol has indicated a good application prospect.

## Conclusions

To the best of our knowledge, the novel type of two-stage breast reconstruction protocol, which includes tissue expansion on the first stage and implant insertion with endoscopy-assisted LDMF transfer on the second stage, is reported for the first time in China. During the process, the use of endoscopic technique and a supplementary posterior lumbar approach allow the procedures to reduce visible scars, avoid the patch effect, while require short time for LDMF harvest and present low incidence of complications. The reported protocol is useful for breast reconstruction because it results in an objectively smaller scar at the donor site while achieves a good outcome of the reconstructed breast according to the evaluation of our patients.

## Data Availability

The datasets used and/or analysed during the current study are available from the corresponding author on reasonable request.

## Abbreviations

LDMF: Latissimus dorsi muscle flap
TE: Tissue expander
3D-SI: Three-dimensional surface image
BMI: Body mass index
